# Less Aggressive Surgical Procedure for Treatment of Solid Pseudopapillary Tumor: Limited Experience from a Single Institute

**DOI:** 10.1371/journal.pone.0143452

**Published:** 2015-11-23

**Authors:** Chi Zhang, Fangfeng Liu, Hong Chang, Hongguang Li, Xu Zhou, Jun Lu, Chengkun Qin, Yongjie Sun, Huidong Sun, Jianbo Lin

**Affiliations:** Department of General Surgery, Shandong Provincial Hospital affiliated to Shandong University, Jinan, Shandong, China; University of Florida, UNITED STATES

## Abstract

**Objectives:**

To evaluate the clinical characteristics and radiological features of solid pseudopapillary tumor (SPT) and assess surgical therapy strategy.

**Methods:**

A retrospective review was performed in 62 patients pathologically confirmed of SPT treated between 2003 and 2014. The clinical features, radiological examinations and surgical strategies were analyzed.

**Results:**

56 females and 6 males were included in this study, mean age was 26 years old (range: 8–66 years old) with mean size of the tumor was 7.2 cm (range: 3–15 cm), and most tumor were commonly located in the head of pancreas (n = 29). Among all the cases, 3 patients had liver metastasis and underwent resection of SPT and liver metastasis. Furthermore, we performed 29 cases of local tumor excision; other patients underwent pancreaticoduodenectomy, middle pancreatectomy, middle pancreatectomy with splenectomy, distal pancreatectomy with spleen preservation, distal pancreatectomy with splenectomy and duodenum-preserving pancreatic head resection. No patient suffered from lymph node metastases. After median follow-up of 46 months (range: 2–135 months), no mortality or local recurrence or distant metastasis was found.

**Conclusions:**

Solid pseudopapillary tumor is a latent malignant tumor with excellent prognosis. If feasible, less aggressive resection without regular lymphadenectomy is recommended for treatment of patients with SPT.

## Introduction

Solid pseudopapillary tumor (SPT) of pancreas is a rare neoplasm that typically occurs in young women, accounting for only 1%–2% of exocrine pancreatic tumors and about 5% of cystic pancreatic tumors [1]. Previously, due to the limited number and indolent course of these tumors, little was known about the natural course of the disease. With the widespread availability of high-quality imaging systems and a better understanding of its pathology, the number of patients diagnosed of SPT increased considerably. This had resulted in a significant change in diagnostic and treatment approaches over the decades.

The clinicopathologic features of SPT are unique: slow-growing, low-grade malignancy [2]. The tumor can be located in any part of the pancreas. Most patients have no specific clinical symptoms or signs and are usually found during routine medical examinations. The growth pattern and metastasis are still incompletely understood. Approximately 10–15% of SPT demonstrate malignant behavior with recurrence and metastasis [3, 4]. The WHO defines tumors with surrounding tissue invasion, perineural invasion, vascular invasion on microscopic pathology, and metastasis as malignant SPT [5]. En bloc resection has been regarded as the only curative treatment. However, there are no definite recommendations regarding the resection extent of primary neoplasm, management of locoregional or distant metastases. Moreover, the necessity of regional lymphadenectomy needs to be delineated, especially in considering of the low malignancy of this tumor [1, 6]. A generally accepted operation algorithm would be desirable as a basic guide for surgical management of SPT. In this study, 62 cases of SPT treated in our hospital were analyzed retrospectively; attempted to summarize the clinical features, surgical strategies and long-term follow up for a better understanding of its natural history and prognosis.

## Materials and Methods

Through a computerized search of medical records, we identified a total of 62 patients who were diagnosed pathologically as pancreatic SPT at the Department of General Surgery, Shandong Provincial Hospital affiliated to Shandong University, from July 2003 to June 2014. The clinical data, radiological findings and surgical interventions were analyzed. In our center, after surgery, patients were followed up after 4 weeks, 6 months, and one year then annually until death or were lost to follow up study. Each visit included a clinical examination, routine laboratory investigation (including complete blood picture, liver function, blood sugar, amylase, and tumor markers CEA, CA19-9), abdominal and pelvic ultrasound, abdominal computed tomography (CT) and Magnetic Resonance Image (MRI) were performed if needed.

Concerning the surgical resection methods, we tended to perform organ-preserving resection to remove SPT if it is possible. For pancreatic tail SPT, if the vessels of the spleen could be preserved, spleen-preserving distal pancreatectomy was attempted in our patients. We practiced the technique of preserving both the splenic artery and vein. The level of resection might commence at the proximal body or the neck, depending on the position of SPT, and proceeded in a retrograde fashion, with preservation of both the splenic artery and vein. After dissection and mobilization, a plane was developed between the splenic, portal or superior mesenteric vein and the pancreas. The splenic artery and vein were slung on a vessel loop and individual vessels supplying or draining the pancreas were meticulously ligated until the dissection reached the splenic hilum. After mobilization was completed, SPT and the adjacent pancreatic body and tail were removed totally, and then the proximal pancreatic transection was over sewn with 5–0 continuous sutures. For SPT tumors located in the body, resection of the pancreas midportion and the tumors with preserving the head and tail portion could be achieved. The main surgical procedures were as follows. Depending on the position of the lesions, the splenic vein and artery were carefully divided away from the pancreas and individual vessels supplying or draining the pancreas were meticulously ligated. The involved pancreatic segment was mobilized on both cephalic and caudal sides. The pancreas with SPT was then dissected. Hemostasis of the stumps of pancreas was performed and the cephalic pancreatic cut surface was closed by 5–0 continuous suture. Finally, the distal side stump was reconstructed by Roux-en-Y pancreaticojejunostomy. Head of the pancreas was common position of SPT. To treat these patients, if the tumors protrude outward the pancreatic tissue, without pancreatic duct compression or adjacency, we recommend local tumor enucleation. In these cases, carefully dissection of SPT from normal surrounding pancreas tissue was performed attempting not to injure the main pancreatic duct. If the main pancreatic duct was not damaged during operation, careful hemostasis of the pancreatic cut surface could be performed using 5–0 continuous sutures. Or else, anastomosis of the pancreatic stump to a Roux-en-Y loop of the jejunum was a rational option. In accordance with our surgical experience, once the SPT located in pancreatic head was more than 5cm, close to or compress the pancreatic duct and local tumor cannot be enucleated, duodenum-preserving pancreatic head resection was another choice other than aggressive pancreatoduodenectomy. Duodenum-preserving pancreatic head resection was first described by Beger et al. in 1985 for treatment of chronic pancreatitis [7]. In accordance with the report, along with resection of the pancreatic head and SPT up to the intrapancreatic common bile duct but preservation of the dorsal capsule of the head and the posterior vessels, then the restoration of the exocrine pancreatic secretory flow from remained pancreatic head and left pancreas into the upper intestinal tract was achieved through interposition of the uppermost jejunal loop (S1 Fig).Pancreatic fistula (PF) was diagnosed when there was measurable drain output on or after postoperative 3 days, with amylase content 3 times the upper limit of normal serum value, according to the International Study Group of Pancreatic Fistula (ISGPF) [8]. Recurrence was defined as SPT detected in pancreas or other organs after first operation. This clinical research was approved by Medical Ethics committee of Shandong University and patient information was anonymized and de-identified prior to analysis.

## Results

### Patient Characteristics

Of the 62 patients diagnosed as SPT, 56 patients (90.3%) were female, the female-to-male ratio of 9.3:1. The mean age at the time of presentation was 26 years (range: 8–66 years). Of the 62 patients, 15 (24.2%) were asymptomatic, with the diagnosis made by occasional ultrasonography (US) or computed tomography (CT). The most common clinical manifestation was abdominal discomfort and pain, which occurred in 29 (46.8%) of the 62 patients; a palpable abdominal mass was presented in 13 (21.0%) patients; 2 patients presented abdominal pain and palpable mass. Jaundice was the main manifestation in 3 patients. 1 patient was detected by CT after trauma. The most frequent location of SPT in our study was pancreatic head (29 cases, 46.8%), followed by tail (21 cases, 33.9%), and the body (12 cases, 19.4%) The mean size of the tumor was 7.2 cm (range: 3–15 cm). And at head, tail and body, the tumor size was 6.4 cm (range: 3–13 cm), 7.8 cm (range: 4–15 cm) and 8.0 cm (range: 3–15 cm), respectively (Table 1 and Table 2).

**Table 1:** Demographic characteristics.

Clinical characteristics	n = 62
Gender	
Female	56
Male	6
Age of presentation (years)	
Mean	26
Range	8–66
Symptomatic	47
abdominal pain	29
palpable abdominal mass	13
abdominal pain and palpable mass	2
jaundice	3
Asymptomatic	15

**Table 2:** Tumor information.

Tumor characteristics	n = 62
Localization	
Head	29
Body	12
Tail	21
Size cm	
Mean	7.2
Range	3–15
Others characteristics	
Liver metastases	3
Lymph node involvement	1

### Preoperative Examination

**Fig 1:**
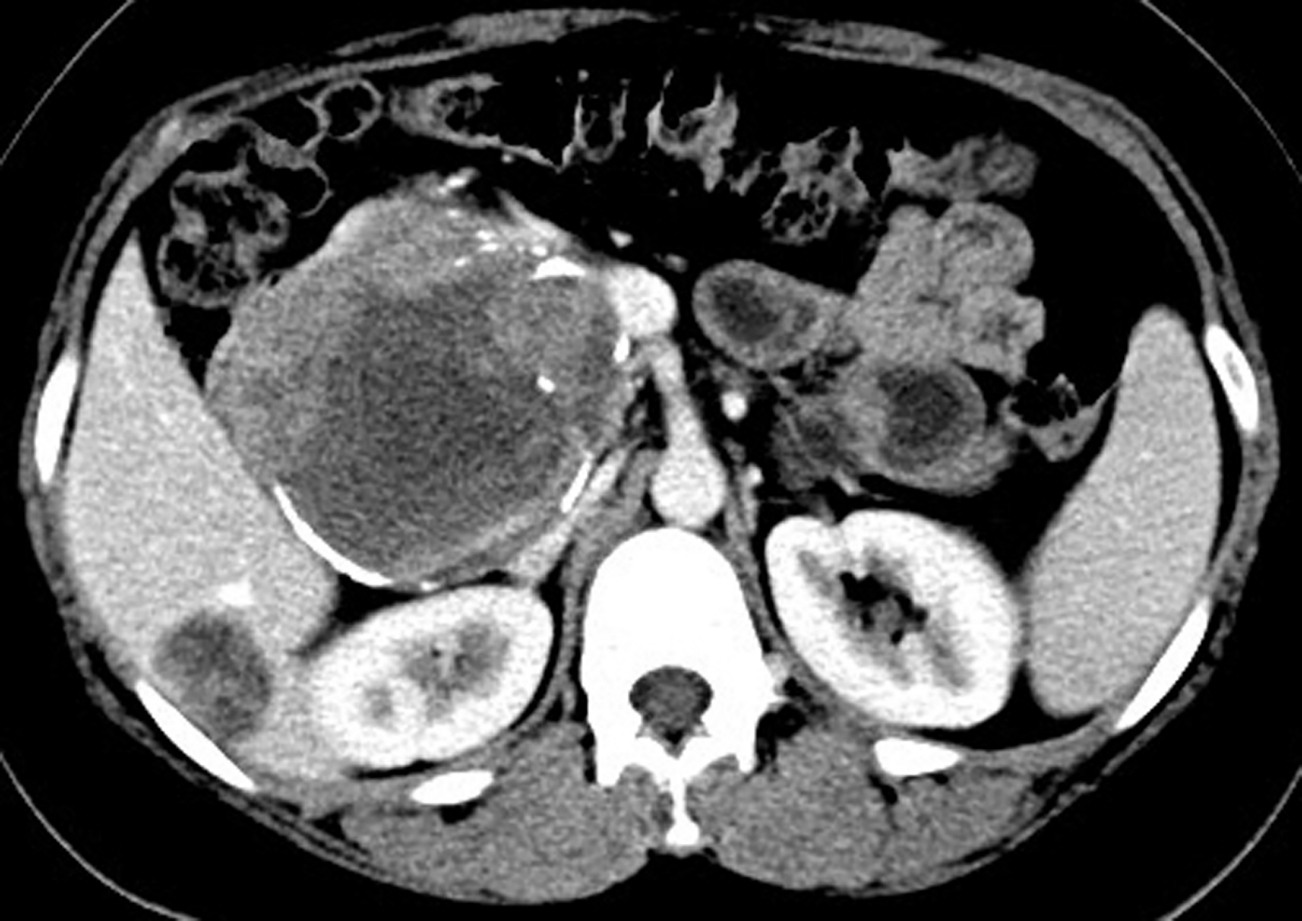
Abdominal CT showing SPT located in head of pancreas and liver metastatic lesion in the right lobe.

Preoperative examination included tumor marker studies, US, CT, or MRI. For all patients, the time interval between imaging examination and histopathological confirmation was less than 15 days. Among the 62 patients of SPT, CT scan was performed in 54 cases and 43 were correctly diagnosed with SPT. US was performed in 41 cases and 25 were correctly diagnosed. What is more, 6 cases accepted MRI, 4 of which were successfully diagnosed. In this study, MRI detected 3 cases of SPT that were not diagnosed by CT and US. Among the 62 patients, 3 cases were found pancreatic neoplasm with liver metastasis by CT scan (Fig 1). The pathological examination verified 3 cases were SPT accompanied with liver metastasis. No enlarged lymph nodes were found by imaging examinations. Tumor markers were always normal in our research; only 3 patients presented a slight elevation of CA 19–9 (41–48 kU/L, the normal value: 0–39Ku/L).

### Surgical Treatment

All the 62 patients underwent complete surgical resection, including 7 cases of pancreaticoduodenectomy, 2 cases of middle pancreatectomy, 1 cases of middle pancreatectomy with splenectomy, 4 cases of distal pancreatectomy with spleen preservation, 13 cases of distal pancreatectomy with splenectomy, 29 cases of local tumor excision, and 3 cases of duodenum-preserving pancreatic head resection. What is more, 1 case underwent enucleation of the local lesion of SPT and liver metastasis. 2 cases underwent distal pancreatectomy with splenectomy plus liver metastasis resection. Of the 29 patients underwent local tumor excision, the mean size was 7.2 cm (range: 3–15 cm) and 18 cases of them located at head, 5 at body, 6 at tail. Of the 3 cases underwent duodenum-preserving pancreatic head resection, the size were 5, 5, 3 cm, respectively. No patient was performed laparoscopically and underwent either chemotherapy or radiotherapy. The overall complication rate was 22.6% (n = 14), with no perioperative deaths.

In our study, Postoperative PF occurred to 3 patients who underwent local tumor excision (2 cases located at body, 1 case located at tail), 2 patients underwent pancreaticoduodenectomy and 1 patient underwent middle pancreatectomy with splenectomy. According to ISGPF criteria [8], 4 cases of PF were divided to Grade A and had fistula without clinical sequelae, 2 cases were divided to Grade B and had fistula requiring therapeutic intervention. PF was treated with percutaneous drainage and medicine for pancreatic secretion inhibition, and they were recovered uneventfully. Abdominal abscess happened to 2 patients who underwent pancreaticoduodenectomy and 2 patients underwent distal pancreatectomy with splenectomy. 1 patient who underwent middle pancreatectomy suffering postoperative PF and 1 patient underwent pancreaticoduodenectomy suffering abdominal abscess also had complication of pulmonary infection and fluid collection. Furthermore, surgical complications included 2 cases of incision infection and 2 cases of urinary tract infection. All the above mentioned cases were treated with drainage and antibiotics accordingly, they were recovered uneventfully (Table 3).

**Table 3:** Characteristics and outcome of surgical management.

Type of pancreatic resection and Complications	n = 62
Type of pancreatic resection	
Pancreaticoduodenectomy	7
Middle pancreatectomy	2
Middle pancreatectomy with splenectomy	1
Distal pancreatectomy with spleen preservation	4
Distal pancreatectomy with splenectomy	13
Local tumor excision	29
Pancreatic head resection with duodenum preservation	3
Enucleation of the local resection of SPT and liver metastasis	1
Distal pancreatectomy with splenectomy plus liver metastasis resection	2
Complications	
Postoperative pancreatic fistula	6
Local tumor excision	3 (50%)
Pancreaticoduodenectomy	2 (33.3%)
Middle pancreatectomy with splenectomy	1 (16.7%)
Abdominal abscess	4
Pancreaticoduodenectomy	2 (50%)
Distal pancreatectomy with splenectomy	2 (50%)
Incision infection	2
Local tumor excision	2 (100%)
Urinary tract infection	2
Pancreaticoduodenectomy	2 (100%)
Pulmonary infection and fluid collection	2
Middle pancreatectomy	1 (50%)
Pancreaticoduodenectomy	1 (50%)

### Pathologic Examination

The mean size of the tumor was 7.2 cm (range: 3–15 cm). Final pathology confirmed the diagnosis of SPT in all patients and complete resection (R0 resection) by two pathologists independently. We dissected the tumor to ensure that no SPT tissue left macroscopically. Besides, the intraoperative pathological frozen slice confirmed the diagnosis of SPT, and no tumor tissue detected microscopically in the margin. The median number of lymph nodes pathologically assessed was 5 (range 0–17 lymph nodes). All dissected enlarged lymph nodes were confirmed to be benign or reactive hyperplasia pathologically. Besides, no lymphovascular invasion, perineural invasion and invasion of surrounding tissue were detected. The pathology of 3 patient's liver metastasectomy specimens shows sheets of bland cells with oval to round nuclei, moderate cytoplasm, ill-defined cell borders, and a focal pseudopapillary appearance. On immunostaining, the tumor cells were positive of CD10, CD56, β-catenin, and progesterone receptor as well asα1-antitrypsin and vimentin. There was focal positivity for synaptophysin. No staining was seen with chromogranin, α1-antichymotrypsin, pan-cytokeratin, galectin-3, and E-cadherin. The histologic appearance and immunohistochemical staining pattern supported a diagnosis of metastatic SPT in the liver. Summing up the above oncologic results, 3 cases in our study presented a malignant SPT according to the WHO standard [5].

### Follow-Up and Survival

The median follow-up period was 46 months (range: 2–135 months). No mortality or local recurrence or distant metastasis was found. And the routine laboratory investigations at follow up visits were all within normal limits.

## Discussions

SPT is a rare neoplasm of low malignant potential [2]. Its pathogenesis still remains unknown. The strong female predilection and the reported expression of progesterone receptors in some cases suggest association between female sex hormones and tumorigenesis, but a causal relationship has not been definitively proved [9]. Recently, genetic research demonstrated SPT is characterized by activating β-catenin gene mutations, which interfere with protein phosphorylation. Translocation of β-catenin regulates the transcription of the growth regulatory genes cyclin D1 and c-myc [10]. Furthermore, β-catenin interacts with E-cadherin, interfering normal cell to-cell interactions [11]. All these theories about the exact origin of SPT are unproved yet, further investigation is needed.

Compared with other pancreatic tumors, SPT usually affect young women in the second or third decade of life [12, 13]. The mean age of patients at diagnosis in our series was 26 years, consistent with several other series [6, 13]. T. Papavramidis summarized 718 cases of SPT and reported the diagnosis was made at a mean age of 22 years (range: 2–85 years old) and there was a tenfold female preponderance [14]. The clinical manifestations of SPT are usually nonspecific; include abdominal discomfort or pain, and/or a palpable mass [15]. In Yunqiang Cai’s study, 115 SPT cases were collected, the symptoms included upper abdominal pain in 46 patients (40.0%), abdominal discomfort in 19 patients (16.5%), and palpable abdominal mass in 17 patients (14.8%) [15]. Among the patients in our study, 75.8% had symptoms, whereas 24.2% were diagnosed incidentally. Pancreatic head and tail were the most common locations of SPT, consistent with other studies. A few cases of SPT have been found adjacent to but anatomically separate from the pancreas, including in the mesocolon, omentum, retroperitoneum, and liver [16]. In our series, one case was found located in mesenteric root adjacent to uncinate process of pancreas.

**Fig 2:**
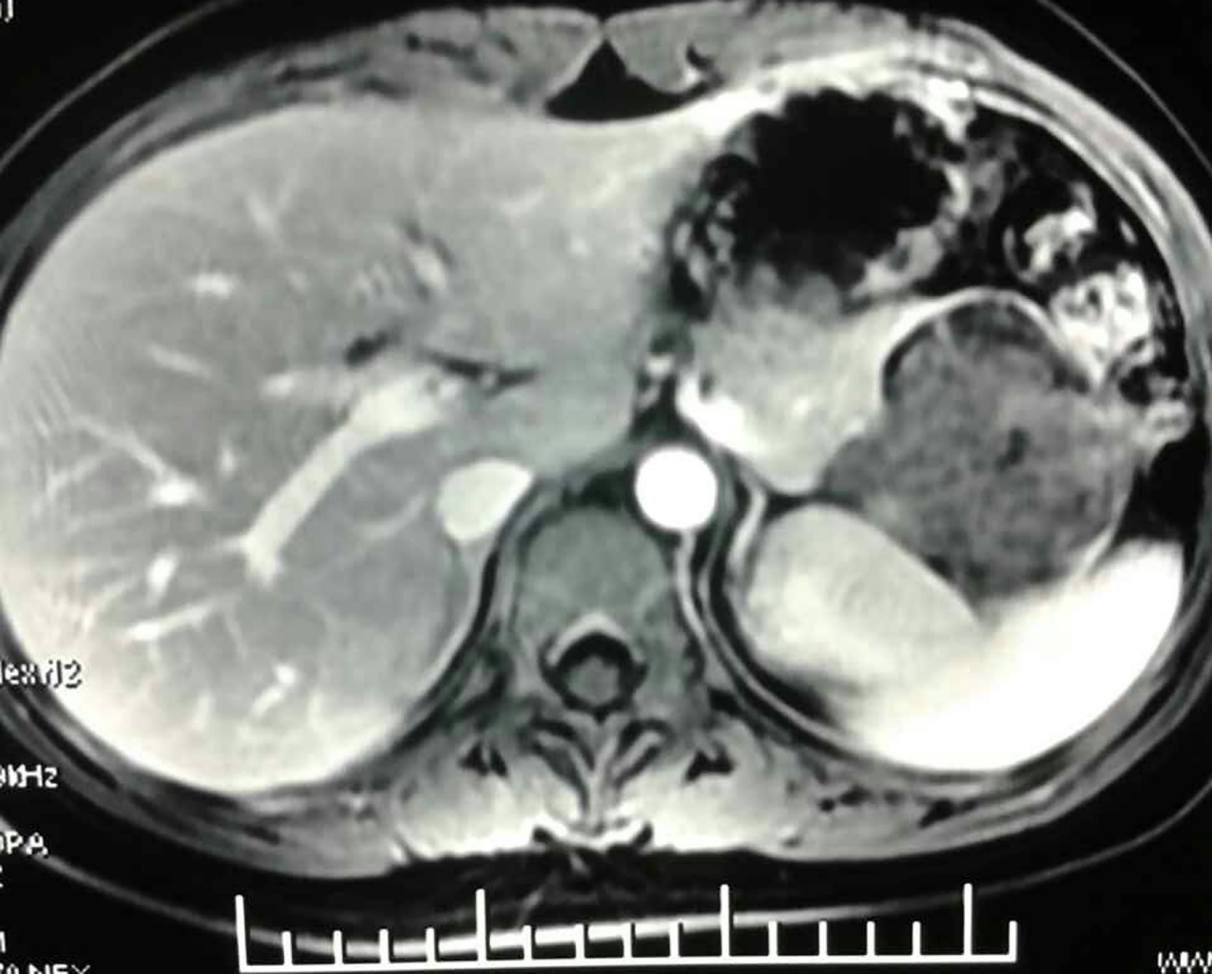
CT examination of SPT shows a well-encapsulated, heterogeneous cystic and solid pancreatic mass.

**Fig 3:**
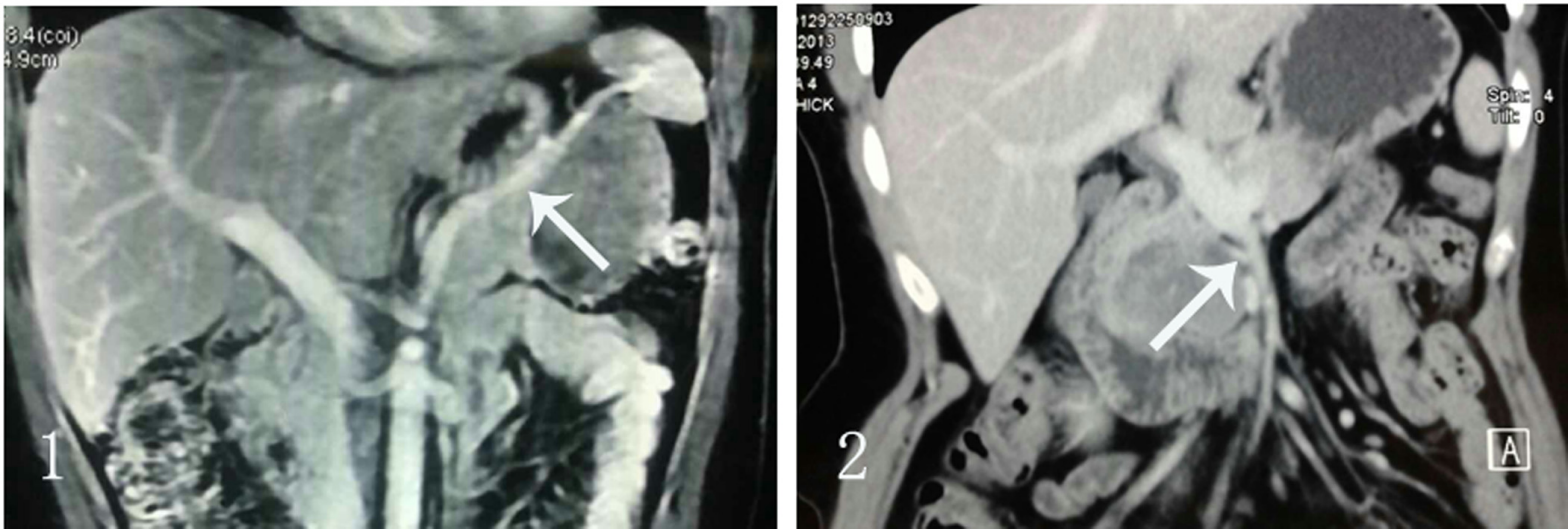
CT scan showed the vessels were jostled by the mass and had clear boundary. 1. The mass had clear boundary with spleen vessels. 2. The mass has clear boundary with SMV.

Advances in imaging strategies have improved the accuracy of the preoperative diagnosis of SPT. The imaging modalities are very useful in preoperative assessment of this disease and could provide strong evidence for treatment protocol planning [17]. Compared with pancreas carcinoma, CT examination of SPT shows a well-encapsulated, heterogeneous cystic and solid pancreatic mass with calcification and hemorrhage, infrequently accompanied with pancreatic duct and bile dilatation (Fig 2). Obstructive jaundice is not a common feature—although in our study most SPT arised from the portion of pancreatic head. In Papavramidis’ series, only 1% of patients with pancreatic head tumors were jaundiced [14]. To our knowledge, lymph node metastasis or enlargement was rarely detected in imaging examination by other studies. Vascular encasement was reported in some cases. In 115 patients of SPT reported by Yunqiang Cai, 32 cases of vessel involvement were found, including 21 cases of splenic vessels and 11 cases of superior mesenteric vein or/and portal vein[15]. Interestingly, in our series no pathological vascular invasion was demonstrated in patients, as either occlusion or narrowing of a vessel by a soft-tissue mass surrounding the area of involvement, which is commonly seen in pancreatic carcinoma. In our study, CT scan showed the adjacent vessels were jostled by the mass and usually had clear boundary (Fig 3). Similarly in surgical exploration, the adjacent arteries and vessels were confirmed to be extracted by the SPT masses, therefore, dissected from the tumor subsequently.

Surgery is the first choice of treatment once the diagnosis of SPT is established, since the overall survival rate after 5 years and 10 years post surgical resection were 95–98% and 93% respectively including patients with malignant SPT [14, 18]. However, the optimal surgical procedures still remain controversial [19–21]. According to the tumor location and invasive potential, pancreatoduodenectomy, distal pancreatectomy (with or without splenectomy), middle pancreatectomy, or enucleation can be performed. Considering the low-grade malignancy nature of this disease and the tumor is usually found in young female patients under 30 years old, it might be reasonable to remove the tumor completely, in the meantime maintain the usual physiological function and anatomy structure by reserving the normal tissue and organs as much as possible. In accordance with such concerns, and the intraoperative pathological frozen slice confirmed low-grade malignant tumor, we tended to perform organ-preserving resection to remove SPT if it is possible. In our previous study, 18 SPT patients were analyzed and the results demonstrated less aggressive surgical resection of the primary lesion achieved equal prognosis compared with aggressive surgery [22]. In Akiyama H’s literature, 35% of SPTs originating in the pancreatic head have been treated with enucleation, and over 60% have been resected by a classic or pylorus-preserving pancreatoduodenectomy [23]. In a retrospective series of 34 patients, Li et al. compared “standard” and “minimized” pancreatic resections for SPT. Both groups had similar morbidity rates and long-term survival, but patients subjected to “standard resections” had longer operating times (225 versus 124 minutes; = 0.004), transfusion rates (53% versus 13%; = 0.03), and hospitalization (21 versus 16 days; = 0.034). Based on these preliminary data, Li et al. advocated “minimized resections, such as enucleation” [24].

Compared with pancreatic cancer, regional lymphatic dissection is not indicated for SPT patients, because incidence of lymph node metastasis is extremely rare [15, 25, 26]. In our series, no patient suffered from lymph node metastases. Among 1723 SPT patients reported by several papers, nodal involvement occurred in only 5 cases [12–15, 20, 26–30]. Thus, routine lymphadenectomy seems not be suggested strongly based on the findings of very low incidence of lymph node metastasis of SPT mentioned above, however macroscopically suspicious enlarged lymph nodes need to be removed. Based on the findings of our series, the indication of lymphadenectomy for SPT patients is needed to be investigated further.

Although the malignant potential of SPT is low, 10–15% of SPT patients develop metastasis. The most common organ of metastasis is liver [31]. It has been suggested that a metastasectomy in the liver with a 1-cm margin could be sufficient based on presumptions from the biological behavior of the tumor [32]. In this study, 3 patients of primary SPT of pancreas were diagnosed liver metastasis by CT scan. One case with primary tumor located in head underwent enucleation of SPT and liver metastasis. The other two cases suffered pancreatic tail SPT underwent distal pancreatectomy with splenectomy plus liver metastasis resection. After follow-up of 74 months, 18 months and 4 months respectively, no recurrence and metastasis occurred to these 3 patients. In Wei-Bin Wang’s study, 187 patients with pancreatic SPT underwent En bloc resection, and liver metastasis occurred in 4 patients. Each patient with metastases underwent surgical resection. After mean follow-up period of 30 months (range, 1–64 months), none of the patients had obvious recurrence or distant metastasis [33]. Therefore, aggressive surgical resection could achieve good prognosis in these patients even in the presence of liver metastasis. For patients of SPT with liver metastasis, if the metastatic lesion is resectable, simultaneous resection of the primary lesion together with the liver metastasis is a reasonable surgical solution.

In conclusion, SPT is a latent malignant tumor that commonly occurs in young female patients before the age of 30. Surgical resection is the main therapeutic strategy and achieves good long-term survival. Considering the excellent prognosis and low-grade malignancy, less aggressive resection of primary SPT, such as spleen-preserving distal pancreatectomy, duodenum-preserving pancreatic head resection and tumor enucleation, is safe and advised. Extensive lymphadenectomy is not necessary due to infrequent nodal metastases. Liver is a common site of SPT metastasis. To treat these patients, synchronous resection of distant metastases and primary tumor is recommended.

## Supporting Information

S1 Fig The postoperative reconstruction of pancreatic head resection with duodenum preservation. (TIF)
