# Supplementary figures and images for: Less Aggressive Surgical Procedure for Treatment of Solid Pseudopapillary Tumor: Limited Experience from a Single Institute
Source: PLoS One. 2015 Nov 23;10(11):e0143452. doi: 10.1371/journal.pone.0143452 (PMC4658154; doi:10.1371/journal.pone.0143452)

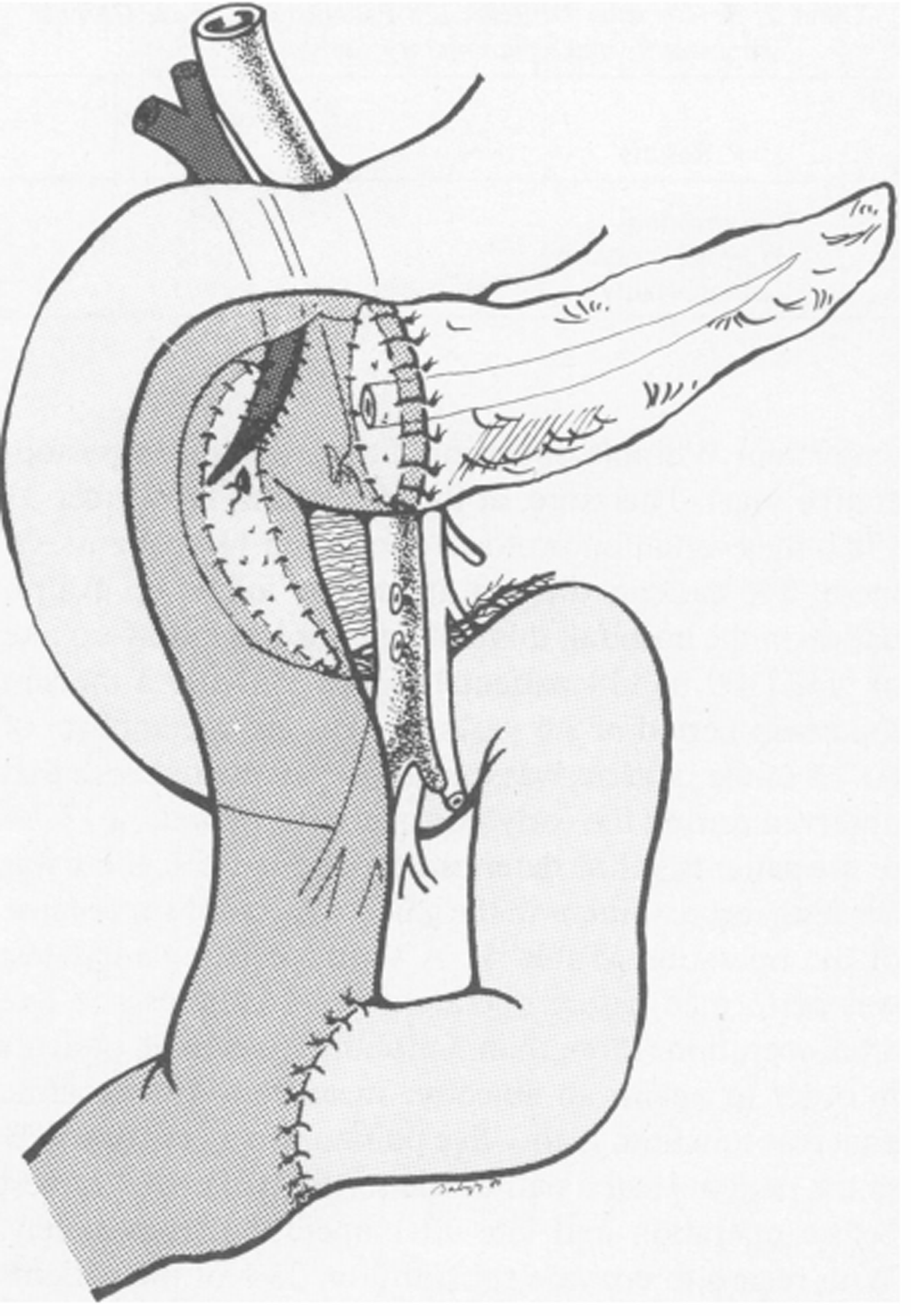

Supplement: S1 Fig — (TIF) [file pone.0143452.s001.tif]
